# Estrogen Signaling through Estrogen Receptor Beta and G-Protein-Coupled Estrogen Receptor 1 in Human Cerebral Vascular Endothelial Cells: Implications for Cerebral Aneurysms

**DOI:** 10.1155/2013/524324

**Published:** 2013-11-12

**Authors:** Jian Tu, Nurul F. Jufri

**Affiliations:** Australian School of Advanced Medicine, Macquarie University, 2 Technology Place, North Ryde, Sydney, NSW 2109, Australia

## Abstract

Little is known about estrogen receptors and their signaling mechanisms in human cerebral vascular endothelial cells, which is important for understanding cerebral aneurysm pathogenesis in menopausal and postmenopausal women. Estrogen receptor beta (ER**β**) and G-protein-coupled receptor 1 (GPER1) were immunocytochemically identified in human cerebral vascular endothelial cells (HCVECs). ER**β** was mainly located at the nuclei of the cells while GPER1 was located at the plasma membrane. Interaction events between 17**β**-estradiol and ER**β** or GPER1 in HCVECs were evaluated by *in situ* proximity ligation assay. The number of interaction events between 17**β**-estradiol and ER**β** was positively correlated with 17**β**-estradiol concentrations (*r* = 0.9614, *P* < 0.01). The interaction events between 17**β**-estradiol and GPER1 were dose responsive. Our data support HCVECs to serve as a suitable cellular model for studying cerebral aneurysm pathogenesis in menopausal and postmenopausal women. Subtypes of estrogen receptors and their signaling mechanisms identified in HCVECs could be applicable for developing estrogen-like compounds to specifically bind to a subtype of estrogen receptors with greater specific action on the cerebral arteries, without the estrogen-dependent side effects on the reproductive organs, to prevent cerebral aneurysm formation in menopausal and postmenopausal woman.

## 1. Introduction

The incidence of cerebral aneurysms is doubled in menopausal and postmenopausal women compared with premenopausal women at the same age [1]. There have been several important studies showing that inflammation underlies cerebral aneurysm formation and rupture [2–4]. A failure of endothelial cells in the cerebral arterial wall to adequately express estrogen receptors abrogates the possibility of direct interaction between estrogen receptors and circulating estrogen, which can trigger inflammation, upregulation of proteolytic pathways, loss of the arterial wall matrix, and degradation of the arterial wall [5, 6]. As no biopsy specimens are possibly obtained during surgery because of high risk of bleeding, there is an increasing interest to develop a cellular model to directly investigate the effect of estrogen deficiency and whether it causes a decline in the function and/or number of estrogen receptors, which may promote inflammation and apoptosis in the endothelial cells of cerebral arterial walls. Since endothelial dysfunction is considered the first step in the pathogenesis of cerebral aneurysms, a human cerebral vascular endothelial cell model was applied in the study.

The arterial protective effects of estrogen act through increased artery compliance, defined as the change in volume for a given change in pressure [7]. Arteries with low compliance less effectively dampen the pulsatile flow of blood by stretching and contracting in response to the systolic and diastolic cardiac phases, respectively. There are three major forms of estrogen, estradiol, estrone, and estriol, in which 17*β*-estradiol has the highest affinity to estrogen receptors. Physiological plasma levels of 17*β*-estradiol range from 118 to 914 pmol/L in premenopausal women and fluctuate in healthy women during the menstrual cycle [8]. The level increases during the follicular phase and gradually declines during the luteal phase of the reproductive cycle. In menopausal and postmenopausal women, estrogen level decreased to less than 73 pmol/L due to reductions in its biosynthesis [9]. 17*β*-Estradiol binds to estrogen receptor alpha (ER*α*) and/or estrogen receptor beta (ER*β*) in the cell's nucleus [10] and mediates long-term genomic effects (hours to days) in remodeling or lipid alteration. It also can bind to G-protein coupled estrogen receptor (GPER) [11] and mediates nongenomic action that is rapid in onset and short in duration such as changes in vasomotor tones through kinase activation and intracellular signaling pathway [12]. The interaction between estrogen and its receptors generates protective effects and maintains homeostasis of the vascular system. Because of alternative RNA splicing, some estrogen receptor isoforms are known to exist. However, exactly which types of estrogen receptors are expressed in human cerebral arterial endothelial cells, their subcellular location, binding characteristics, and functional selectivity remain unknown. The objectives of this study were to examine whether estrogen receptors would present in human cerebral vascular endothelial cells. If so, which types of receptors? How does estrogen interact with its receptors? 

## 2. Materials and Methods

### 2.1. Chemicals, Antibodies, and Cell Culture

All chemicals, M199 media with or without phenol red, fetal bovine serum (FBS), penicillin-streptomycin, and 17*β*-estradiol were purchased from Sigma-Aldrich, Aldrich (St. Louis, MO, USA), unless otherwise specified. Charcoal stripped FBS was purchased from Invitrogen (Carlsbad, CA, USA). Rabbit anti-human monoclonal estrogen receptor beta (ER*β*) antibody was purchased from Novus Biologicals (Littleton, CO, USA). Goat anti-human polyclonal 17*β*-estradiol antibody was purchased from Fitzgerald Industries International (Acton, MA, USA). Rabbit anti-human polyclonal G-protein-coupled estrogen receptor 1 was purchased from MBL International Corporation (Woburn, MA, USA). Alexa-594 conjugated secondary goat anti-rabbit and Alexa-594 conjugated secondary donkey anti-goat antibodies were purchased from Molecular Probe (Eugene, OR, USA). All reagents used for the *in situ* proximity ligation assay (PLA) were purchased from Olink Bioscience (Uppsala, Sweden). Simian virus 40  T antigen immortalized human cerebral vascular endothelial cells (HCVECs) were purchased from Applied Biological Materials (Richmond, BC, Canada) and maintained in M199 medium supplemented with endothelium growth factor, 10% FBS, and 1% penicillin-streptomycin in a humidified atmosphere of 37°C and 5% CO_2_. The genotype and phenotype of HCVECs are stable within 150 passages. The cells used in this study were passages 6–12. Nunc Lab-Tek Chamber slides were purchased from ProSciTech (QLD, Australia).

### 2.2. Immunocytochemistry

Immunocytochemistry was performed as previously described [13]. Briefly, HCVECs were seeded and grown in chamber slides until 50% confluence in M199 phenol red free media supplemented with endothelium growth factor, 10% FBS, and 1% penicillin-streptomycin in a humidified atmosphere of 37°C and 5% CO_2_. The cells were washed twice in PBS (pH 7.2) before being fixed using 4% paraformaldehyde for 15 minutes. The cells were rinsed with PBS and permeabilized in 0.1% Triton X-100 and 0.5% BSA in PBS at room temperature for 15 minutes. After washing with PBS, the cells were blocked in 5% BSA in PBS for 1 hour and incubated with anti-ER*β* or anti-GPER1 antibody (1 : 250) in 5% BSA at room temperature for 2 hours. After washing with PBS-Tween (PBST), the cells were incubated with Alexa-594 conjugated secondary antibody (1 : 800) in 5% BSA at room temperature in the dark for 1 hour. The cells were washed with PBST before being cover-slipped and examined using a confocal microscope (Leica SP5, Germany), and imaging data were analyzed using Leica LAS AF software. Parallel negative controls were performed without primary or secondary antibody. Isotype controls were performed using rabbit IgG to test the specificity of primary antibodies. Each staining was triplicated and repeated 3 times.

### 2.3. Proximity Ligation Assay

Proximity ligation assay was applied to examine the interaction events between ER*β* or GPER1 and 17*β*-estradiol. Two antibodies, one anti-ER*β* or GPER1 and another anti-17*β*-estradiol, covalently linked to oligonucleotide mediate amplifiable DNA molecules. If the receptor and its ligand are in close proximity of interaction, the oligonucleotide would direct the formation of a circular DNA molecule before being amplified, a process known as rolling circle amplification. Red fluorescent tagged probes are introduced and can bind to the rolling circle amplification, resulting in DNA replication and signal production as red dots. Each fluorescent red dot would represent one molecular interaction between ER*β* or GPER1 and 17*β*-estradiol. The *in situ* PLA was performed according to the manufacturer's instructions. Briefly, HCVECs were seeded and grown in chamber slides in M199 media without phenol red until 50% confluence. After washing twice with PBS, the cells were incubated with fresh M199 without phenol red for 3 hours and then treated with 17*β*-estradiol at a concentration of 0, 120, 240, 480, 720, or 960 pmol/L for 24 hours. The cells were washed with PBS, fixed using 4% paraformaldehyde, permeabilized in 0.1% Triton X-100 and 0.5% BSA in PBS, blocked in 10% BSA in PBS, and incubated with a mixture of monoclonal ER*β* antibody or GPER1 antibody and polyclonal 17*β*-estradiol antibody at 1 : 250 dilution at room temperature for 2 hours. Duolink anti-goat PLA PLUS and anti-rabbit PLA MINUS secondary probes were diluted at 1 : 5 in 5% BSA and incubated with the cells at 37°C in a humidified chamber for 1 hour. The ligation and amplification steps for PLA were performed as suggested by Olink using 40 *μ*L volumes. The cells were washed using Olink buffer A and incubated in ligation-ligase solution, containing oligonucleotides that hybridise the PLA probe, at 37°C in a humidity chamber for 30 minutes. The cells were washed using Olink buffer A and incubated in amplification-polymerase solution, containing oligonucleotides probes with red colour fluorophores and polymerase, at 37°C in a humidity chamber for 100 minutes for rolling cycle amplification. The cells were washed using Olink buffer B and stained with DAPI (1 *μ*g/mL) for 1 minute before washing with 0.01% buffer B. The cells were dried in the dark and cover-slipped using Vectashield HardSet Mounting Medium. Fluorescent images were obtained using a confocal microscope (Leica TCS SP5X, Wetzlar, Germany).

Images captured for PLA events were analyzed using Leica LAS AF software (Version 2.4.1; Leica Microsystems GmbH., Wetzlar, Germany). The number of PLA signals was counted from 10 *Z*-plane images. At least 100 cells per condition were quantified using ImageJ analysis. The positive PLA events were observed as fluorescent particles (size from 2 to 50 pixels in diameter). When PLA events merged to create particles larger than 50 pixels, the area was measured, and number of events was assumed to be particle area divided by 10 since 10 pixels were the median size of most PLA events. A threshold of 100 (gray values) was set for a positive signal prior to signal counting. 

### 2.4. Data Analysis

Data were expressed as mean ± SEM (number of experiments). Statistical difference between groups was determined using the unpaired two-tailed *t*-test. When there were more than two groups, differences were analyzed using analysis of variance if the variances were equal and the Mann-Whitney nonparametric test if variances were unequal [14]. Linear regressions were calculated using a statistical computer package, Number Cruncher Statistical Systems [14]. A value of *P* < 0.05 was considered statistically significant.

## 3. Results

### 3.1. Subcellular Localization of ER*β* and GPER1 in Human Cerebral Vascular Endothelial Cells

**Figure 1:**
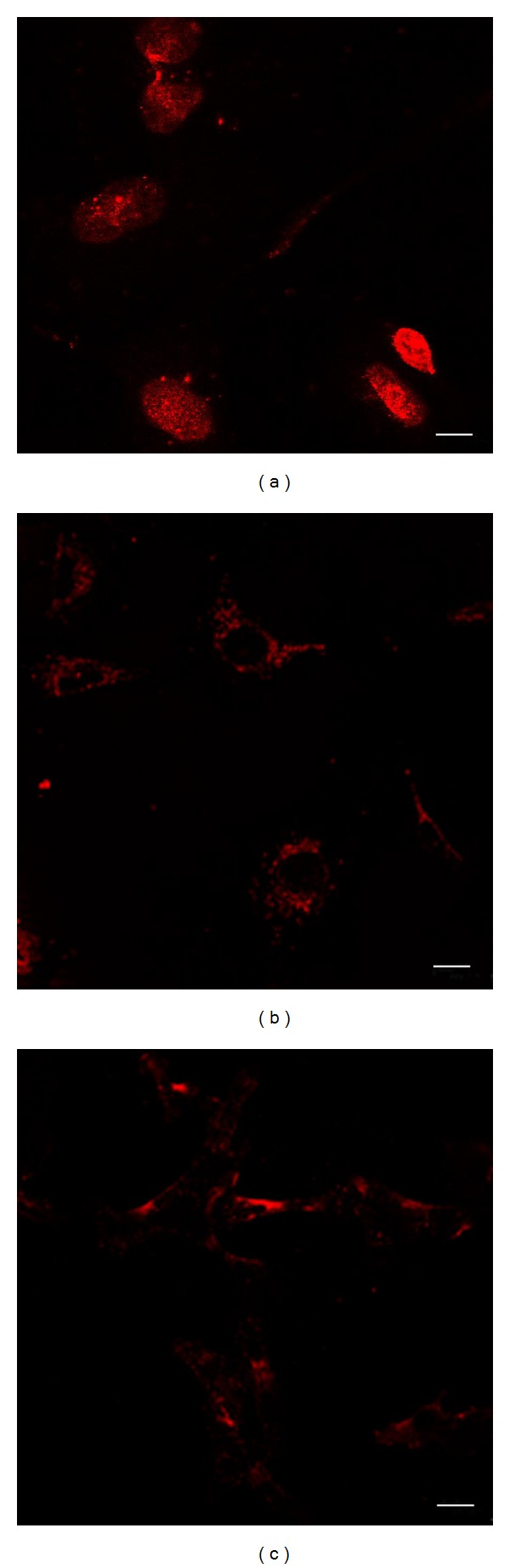
Localization of ER*β* and GPER in human cerebral vascular endothelial cells. (a) ER*β* was immunocytochemically stained in red by a rabbit monoclonal antiestrogen receptor beta antibody and Alexa-594 conjugated secondary antibody at the nuclei of the cells. (b) GPER was immunocytochemically identified in red by anti-G-protein-coupled estrogen receptor antibody and Alexa-594 conjugated secondary antibody at the cytoplasm surrounding the nuclei of the cells when the cell membrane was permeabilized by Triton X-100. Low density of GPER was observed at the nuclei of the cells. (c) GPER signals were dispersed around the plasma membrane of the cells when the cells were not permeabilized. Immunocytochemistry, bar = 10 *μ*m.

High density of immunopositive ER*β* signals was observed at the nuclei of HCVECs (Figure 1(a)), suggesting the expression of ER*β* at the nuclei of human cerebral vascular endothelial cells. There was no positive ER*β* signal identified at the cytoplasm of the cells. In order to differentiate the subcellular location of GPER1, the cells were treated with Triton X-100 to increase membrane permeability or without Triton X-100. When the cells were permeabilized, immunopositive GPER1 signals were dispersed throughout the cytoplasm of HCVECs (Figure 1(b)). There were low density GPER1 signals observed at the nuclei of the cells (Figure 1(b)). When the cells were not permeabilized, GPER1 signals were observed on the cell surface (Figure 1(c)), suggesting the plasma membrane location of GPER1 in human cerebral vascular endothelial cells.

### 3.2. Interaction between ER*β* and 17*β*-Estradiol in Human Cerebral Vascular Endothelial Cells

**Figure 2:**
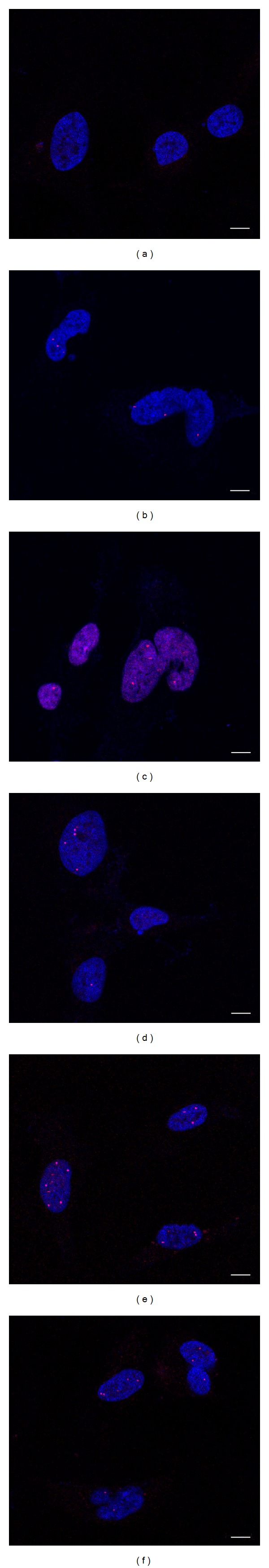
The interaction events between ER*β* and 17*β*-estradiol in human cerebral vascular endothelial cells. The interaction events between ER*β* and 17*β*-estradiol were identified by *in situ* proximity ligation assay in the nuclei of the cells. The nuclei of the cells were positively stained by DAPI in blue. The interaction events between ER*β* and 17*β*-estradiol were identified in red dots in the nuclei of the cells when the cells were exposed to the concentration of 17*β*-estradiol at 0 (a), 120 (b), 240 (c), 480 (d), 720 (e), or 960 pmol/L (f). *In situ* proximity ligation assay, bar = 10 *μ*m.

PLA was applied to examine the interaction events between ER*β* and 17*β*-estradiol in HCVECs. When 17*β*-estradiol was absent from the cell culture, there was no interaction event observed between ER*β* and 17*β*-estradiol (Figure 2(a)), which represents an estrogen deficient condition in HCVECs. When HCVECs were exposed to physiological concentrations of 17*β*-estradiol, proximity ligation revealed the interaction events between ER*β* and 17*β*-estradiol at the nuclei of HCVECs (Figures 2(b)–2(f)), suggesting physiological responsiveness of the cells to 17*β*-estradiol.

### 3.3. Dose Response of Interaction Events between ER*β* and 17*β*-Estradiol in Human Cerebral Vascular Endothelial Cells

**Figure 3:**
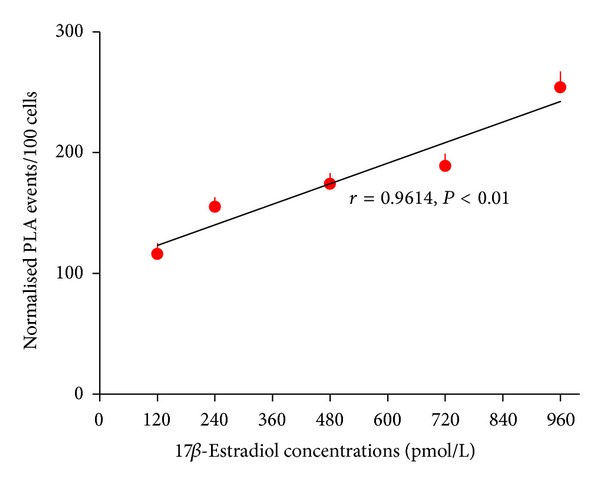
The dose-response curve of interaction events between ER*β* and 17*β*-estradiol in human cerebral vascular endothelial cells. When the cells were exposed to the increasing concentrations of 17*β*-estradiol from 120 to 960 pmol/L, the interaction events between ER*β* and 17*β*-estradiol were increased (*r* = 0.9614, *P* < 0.01). At least 100 cells per concentration were quantified using ImageJ software. The positive PLA events were observed as red fluorescent particles (sizes from 2 to 50 pixels in diameter). When PLA events merged to create particles larger than 50 pixels, the area was measured, and number of events was assumed to be particle area divided by 10 since 10 pixels were the median size of most PLA events. Data were expressed as mean ± SEM of 3 separate experiments.

Proximity ligation revealed that the number of interaction events between ER*β* and 17*β*-estradiol at the nuclei of HCVECs increased with increasing physiological concentrations of 17*β*-estradiol in the cell culture, ranging from 120 to 960 pmol/L (Figures 2 and 3). There was a positive correlation between physiological concentrations of 17*β*-estradiol and the number of interaction events between ER*β* and 17*β*-estradiol in HCVECs (*r* = 0.9614, *P* < 0.01).

### 3.4. Interaction between GPER1 and 17*β*-Estradiol in Permeabilized Human Cerebral Vascular Endothelial Cells

**Figure 4:**
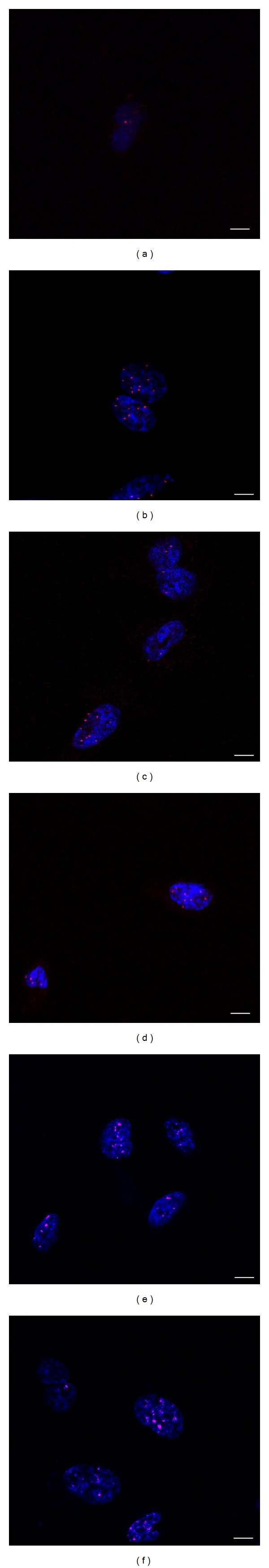
The interaction events between GPER and 17*β*-estradiol in permeabilized human cerebral vascular endothelial cells. The interaction events between GPER and 17*β*-estradiol were identified in the nuclei of the cells. The nuclei of the cells were positively stained by DAPI in blue. The interaction events between GPER and 17*β*-estradiol were identified in red dots when the cells were exposed to the concentration of 17*β*-estradiol at 0 (a), 120 (b), 240 (c), 480 (d), 720 (e), or 960 pmol/L (f). *In situ* proximity ligation assay, bar = 10 *μ*m.

When the plasma membrane of HCVECs was permeabilized by Triton X-100 and exposed to the cell culture condition that 17*β*-estradiol was absent, there was no interaction event identified between GPER1 and 17*β*-estradiol (Figure 4(a)), which represents an estrogen deficient condition in permeabilized HCVECs. When permeabilized HCVECs were exposed to physiological concentrations of 17*β*-estradiol, proximity ligation revealed the interaction events between GPER1 and 17*β*-estradiol at the cytoplasm and nuclei of permeabilized HCVECs (Figures 4(b)–4(f)), suggesting physiological responsiveness of permeabilized HCVECs to 17*β*-estradiol. PLA signals in Figures 4(b)–4(f) were an overlayer of 10 *Z*-plane images, which showed greater number of PLA signals in the nuclei.

### 3.5. Dose Response of Interaction Events between GPER1 and 17*β*-Estradiol in Permeabilized Human Cerebral Vascular Endothelial Cells

**Figure 5:**
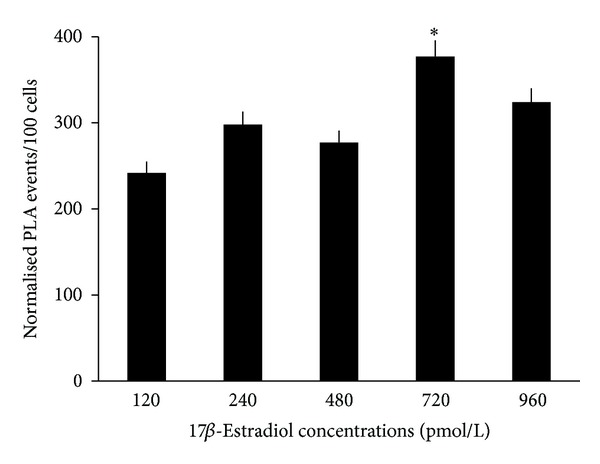
Dose responsiveness of interaction events between GPER and 17*β*-estradiol in permeabilized human cerebral vascular endothelial cells. When the cells were exposed to different concentrations of 17*β*-estradiol ranging from 120 to 960 pmol/L, the interaction events between GPER and 17*β*-estradiol responded to the corresponding dose of 17*β*-estradiol. *P < 0.05 versus the number of interaction signals at other concentrations of 17*β*-estradiol. At least 100 cells per condition were quantified using ImageJ software. The positive PLA signals were observed as red dots (sizes from 2 to 50 pixels in diameter). When PLA events merged to create particles larger than 50 pixels, the area was measured, and number of events was assumed to be particle area divided by 10 since 10 pixels were the median size of most PLA events. Data were expressed as mean ± SEM of 3 separate experiments.

Compared with the number of interaction events between GPER1 and 17*β*-estradiol in permeabilized HCVECs cultured at 120 pmol/L of 17*β*-estradiol, the number of interaction events between GPER1 and 17*β*-estradiol at the nuclei of permeabilized HCVECs increased by 23%, 14%, 56%, and 34% at 240, 480, 720, and 960 pmol/L of 17*β*-estradiol, respectively (Figure 5). The highest number of interaction events between GPER1 and 17*β*-estradiol was observed when permeabilized HCVECs were exposed to 720 pmol/L of 17*β*-estradiol.

## 4. Discussion

A human cerebral vascular endothelial cell model has been established for the study of cerebral aneurysm formation. This study has identified ER*β* and GPER1 in HCVECs, confirmed their subcellular locations, verified functional interaction between ER*β* and 17*β*-estradiol or GPER1 and 17*β*-estradiol, and established a positive correlation between physiological dose responsiveness and the interaction events between ER*β* and 17*β*-estradiol or GPER1 and 17*β*-estradiol in HCVECs. This human cerebral vascular endothelial cell model presents implications of estrogen signalling through ER*β* and GPER1 for cerebral aneurysm pathogenesis in menopausal and postmenopausal women. These *in vitro* alterations may provide the foundation for further assessment during *in vivo* assessment.

### 4.1. Estrogen Signaling through ER*β*

Estrogen receptor *α* has previously been identified in rat cerebral blood vessels [15], but there is no report of its expression in human cerebral vascular endothelial cells. This study found that ER*β* was located in the nuclei of human cerebral vascular endothelial cells. The interaction events between 17*β*-estradiol and ER*β* were dose dependent and occurred in the nuclei of the cells. These findings provide evidence that estrogen induced effects on the cell's gene expression are mediated via ER*β*. In the absence of estrogen molecules, ER*β* is inactive and has no influence on gene expression. However, when estrogen molecules enter HCVECs and pass into their nuclei, the estrogen molecules bind to ER*β*, thereby causing the shape of ER*β* to change. This estrogen-ER*β* complex then binds to estrogen response elements, which are located near genes that are controlled by estrogen [16]. After it has become attached to estrogen response elements in DNA, this estrogen-ER*β* complex binds to coactivator proteins and the promoter region of estrogen-responsive genes becomes active, resulting in recruitment of coregulatory proteins to the promoter. The active genes produce mRNA molecules, which guide the synthesis of specific proteins [17]. These proteins can then influence the behaviour of HCVECs.

**Figure 6:**
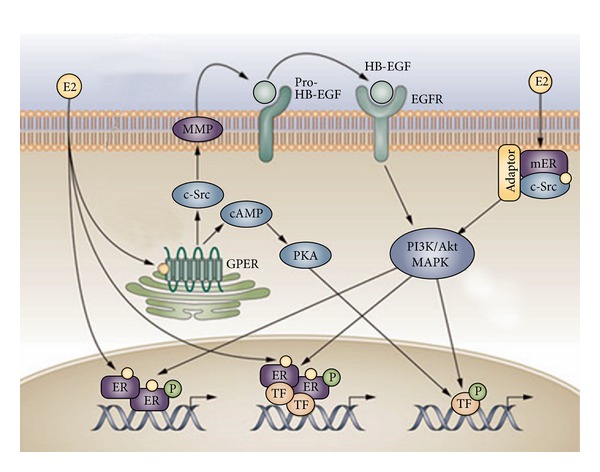
Hypothetical estrogen signaling pathways in human cerebral vascular endothelial cells. 17*β*-Estradiol (E2) passes through plasma and nuclear membranes activate nuclear ER*β* through 3 mechanisms. First mechanism: 17*β*-estradiol causes ER*β* dimerization, binds the ER*β* dimer to the promoter of the estrogen-responsive gene, phosphorylation (P) of ER*β*, and transcriptionally regulates the estrogen-responsive gene. The active genes produce mRNA molecules, which guide the synthesis of specific proteins. These proteins can then influence the behaviour of HCVECs. Second mechanism: activated ER*β* modulates the function of transcription factors (TF) through protein-protein interactions. Third mechanism: 17*β*-estradiol (E2) binds to ER*β* at the plasma membrane (mER). This estrogen-ER*β* complex binds to adaptor 1 (Adaptor) protein and the signaling molecule such as c-Src, which mediates rapid signaling via PI3K-Akt and MAPK pathway to activate the promoter region of estrogen-responsive genes. Alternatively, 17*β*-estradiol (E2) passes through plasma, binds to GPER1 (GPER), and activates c-Src. c-Src activates matrix metalloproteinase (MMP). MMP cleaves pro-heparin-binding-epidermal growth factor (pro-HB-EGF) that transactivates epidermal growth factor receptor (EGFR). EGFR activates phosphatidylinositol 3-kinase-protein kinase B (PI3K-Akt) and mitogen-activated protein kinase (MAPK) pathway. 17*β*-Estradiol (E2) GPER complex also activates cAMP production to restore EGF-activated MAPK to basal levels through protein kinase A (PKA) dependent inhibition of Raf-1 activity. Akt: protein kinase B; c-Src: protooncogene tyrosine-protein kinase Src; E2: 17*β*-estradiol; ER: estrogen receptor beta and/or G-protein-coupled estrogen receptor-1; EGFR: epidermal growth factor receptor; GPER: G-protein-coupled ER; MAPK: mitogen-activated protein kinase; mER: plasma membrane ER; MMP: matrix metalloproteinase; P: phosphorylation; PI3K: phosphatidylinositol 3-kinase; PKA: protein kinase A; pro-HB-EGF: pro-heparin-binding-epidermal growth factor; Raf-1: RAF protooncogene serine/threonine-protein kinase; TF: transcription factors. JT modified the image by permission from Macmillan Publishers Ltd. Nature Reviews Endocrinology [17]. Copyright 2011 to Jian Tu for reuse of the original image from Nature Publishing Group (the original image is available in colour at http://www.nature.com/nrendo/index.html).

More specifically, the activated nuclear ER*β* functions through the following 3 mechanisms (Figure 6) [17]. First, 17*β*-estradiol causes ER*β* dimerization. The ER*β* dimer binds to the promoter of the estrogen-responsive gene, phosphorylation of ER*β*, and transcriptionally regulates the estrogen-responsive gene. The active genes produce mRNA molecules, which guide the synthesis of specific proteins. These proteins can then influence the behaviour of HCVECs. Second, activated ER*β* modulates the function of transcription factors through protein-protein interactions. Third, 17*β*-estradiol binds to ER*β* at the plasma membrane. This estrogen-ER*β* complex binds to adaptor 1 protein and signaling molecule such as c-Src, which mediates rapid signaling via PI3K-Akt and MAPK pathway to activate the promoter region of estrogen-responsive genes. 

### 4.2. Estrogen Signaling through GPER1

The estrogen-ER*β* signaling pathway is not the only signaling pathway in the estrogen signaling network since GPER1 is identified in the same cell. GPER1 may play an important role in estrogen downstream signaling pathway. GPER1 is a transmembrane protein that generates rapid action compared to nuclear receptor ER*β*. GPER1 is situated on the plasma membrane [18] and is also located at endoplasmic reticulum which is continuous with the nuclear membrane [19]. Although the majority of GPER1 is expressed in the plasma membrane, some of it also is functionally expressed at the nuclei of the cells [20]. GPER1 can be translocated to the cell surface or endoplasmic reticulum or nuclei when plasma membrane is permeabilized [21]. When estrogen molecules bind to GPER1, it rapidly activates protein signaling cascade of events via pathways involving G-proteins and stimulating second messenger cAMP production [22], expression of Bcl-2 [23], nerve growth factor [24], cyclin D2 [25], and c-Fos [26]. GPER1 signaling through a pertussis toxin-sensitive G-protein triggers cleavage of membrane-tethered heparin-bound epidermal growth factor (EGF), resulting in transactivation of the EGF receptor [27], intracellular Ca^2+^ mobilization [19, 28], ERK1/2 activation [29], Src activation [11, 30], and PI3K activation (Figure 6) [19]. GPER also regulates transcriptional activity by activating signaling pathways that involve cAMP, ERK, PI3K, and c-Fos (Figure 6) [31]. The distinct mechanism of estrogen-GPER1 signaling is through protein-protein interactions to regulate cellular function.

This study demonstrates that 17*β*-estradiol can simultaneously activate ER*β* and GPER1 at different subcellular locations of HCVECs. Interaction between ER*β* and 17*β*-estradiol triggers a genomic signaling cascade of events, resulting in significant increases in cellular structural protein biosynthesis in HCVECs [32]. There is a positive correlation between dose responsiveness of 17*β*-estradiol and its signaling through ER*β*. Interaction between GPER1 and 17*β*-estradiol initiates a nongenomic signaling cascade of events, resulting in significant increases in regulatory protein biosynthesis in HCVECs (our unpublished data). The number of interaction events depends on the availability of estrogen molecules. Both ER*β* and GPER1 are important in 17*β*-estradiol mediated effects in HCVECs. Estrogen maintains the normal homeostatic process of HCVECs by stimulating endothelial proliferation and reducing vascular tone via its ER*β* and GPER1 [15, 33]. This supports the concept that application of estrogen is beneficial in preserving the normal function of the vascular system [33, 34]. The types of ERs and their signaling mechanisms identified in HCVECs could potentially be applied for developing estrogen-like compounds to specifically bind to a subtype of ERs with greater specific action on the cerebral arteries, without the estrogen-dependent side effects on the reproductive organs. 

## 5. Conclusions 

This study has identified and functionally characterized estrogen signaling through ER*β* and GPER1 in HCVECs. This human cerebral vascular endothelial cell could serve as a cellular model for studying cerebral aneurysm pathogenesis in menopausal and postmenopausal women. Subtypes of ERs and their signaling mechanisms identified in HCVECs could be applicable for developing estrogen-like compounds to specifically bind to a subtype of ERs with greater specific action on the cerebral arteries, without the estrogen-dependent side effects on the reproductive organs, to prevent cerebral aneurysm formation in menopausal and postmenopausal woman.

## Supplementary Material

Nature Pubilshing Group License Terms and Conditions

## Abbreviations

c-Src: Protooncogene tyrosine-protein kinase Src
DAPI: 4,6-Diamidino-2-phenylindole
E2: 17*β*-Estradiol
EGFR: Epidermal growth factor receptor
ER*β*: Estrogen receptor beta
FBS: Fetal bovine serum
HCVECs: Human cerebral vascular endothelial cells
MAPK: Mitogen-activated protein kinase
mER: Plasma membrane ER
MMP: Matrix metalloproteinase
P: Phosphorylation
PGER: G-protein-coupled estrogen receptor 1
PI3K-Akt: Phosphatidylinositol 3-kinase-protein kinase B
PKA: Protein kinase A
PLA: Proximity ligation assay
pro-HB-EGF: Pro-heparin-binding-epidermal growth factor
Raf-1: RAF protooncogene serine/threonine-protein kinase
TF: Transcription factors.
