# Supplementary material for: Estrogen Signaling through Estrogen Receptor Beta and G-Protein-Coupled Estrogen Receptor 1 in Human Cerebral Vascular Endothelial Cells: Implications for Cerebral Aneurysms
Source: Biomed Res Int. 2013 Nov 12;2013:524324. doi: 10.1155/2013/524324 (PMC3844273; doi:10.1155/2013/524324)
Supplement: Supplementary file 1 — Nature Pubilshing Group License Terms and Conditions [file 524324.f1.pdf]

NATURE PUBLISHING GROUP LICENSE
TERMS AND CONDITIONS

Aug 13, 2013

This is a License Agreement between Jian Tu ("You") and Nature Publishing Group ("Nature Publishing Group") provided by Copyright Clearance Center ("CCC"). The license consists of your order details, the terms and conditions provided by Nature Publishing Group, and the payment terms and conditions.

All payments must be made in full to CCC. For payment instructions, please see information listed at the bottom of this form.

License Number	3207030639358
License date	Aug 13, 2013
Licensed content publisher	Nature Publishing Group
Licensed content publication	Nature Reviews Endocrinology
Licensed content title	The G-protein-coupled estrogen receptor GPER in health and disease
Licensed content author	Eric R. Prossnitz and Matthias Barton
Licensed content date	Dec 1, 2011
Volume number	7
Issue number	12
Type of Use	reuse in a journal/magazine
Requestor type	academic/university or research institute
Format	electronic
Portion	figures/tables/illustrations
Number of figures/tables/illustrations	1
High-res required	no
Figures	Figure 2
Author of this NPG article	no
Your reference number	
Title of the article	Estrogen signaling through estrogen receptor-beta and G-protein-coupled estrogen receptor-1 in human cerebral vascular endothelial cells: a cellular model for cerebral aneurysm in postmenopausal women
Publication the new article is in	BioMed Research International
Publisher of your article	hindawi
Author of the article	Nurul F. Jufri, Jian Tu
Expected publication date	Jan 2014

Estimated size of new article (number of pages)	6
Total	0.00 USD

Terms and Conditions

Terms and Conditions for Permissions

Nature Publishing Group hereby grants you a non-exclusive license to reproduce this material for this purpose, and for no other use,subject to the conditions below:

1. NPG warrants that it has, to the best of its knowledge, the rights to license reuse of this material. However, you should ensure that the material you are requesting is original to Nature Publishing Group and does not carry the copyright of another entity (as credited in the published version). If the credit line on any part of the material you have requested indicates that it was reprinted or adapted by NPG with permission from another source, then you should also seek permission from that source to reuse the material.

2. Permission granted free of charge for material in print is also usually granted for any electronic version of that work, provided that the material is incidental to the work as a whole and that the electronic version is essentially equivalent to, or substitutes for, the print version.Where print permission has been granted for a fee, separate permission must be obtained for any additional, electronic re-use (unless, as in the case of a full paper, this has already been accounted for during your initial request in the calculation of a print run).NB: In all cases, web-based use of full-text articles must be authorized separately through the 'Use on a Web Site' option when requesting permission.

3. Permission granted for a first edition does not apply to second and subsequent editions and for editions in other languages (except for signatories to the STM Permissions Guidelines, or where the first edition permission was granted for free).

4. Nature Publishing Group's permission must be acknowledged next to the figure, table or abstract in print. In electronic form, this acknowledgement must be visible at the same time as the figure/table/abstract, and must be hyperlinked to the journal's homepage.

5. The credit line should read:
   Reprinted by permission from Macmillan Publishers Ltd: [JOURNAL NAME] (reference citation), copyright (year of publication)
   For AOP papers, the credit line should read:
   Reprinted by permission from Macmillan Publishers Ltd: [JOURNAL NAME], advance online publication, day month year (doi: 10.1038/sj.[JOURNAL ACRONYM].XXXXX)

<u>Note: For republication from the *British Journal of Cancer*, the following credit lines apply.</u>
Reprinted by permission from Macmillan Publishers Ltd on behalf of Cancer Research UK: [JOURNAL NAME] (reference citation), copyright (year of publication)For AOP papers, the credit line should read:
Reprinted by permission from Macmillan Publishers Ltd on behalf of Cancer Research UK: [JOURNAL NAME], advance online publication, day month year (doi: 10.1038/sj.[JOURNAL ACRONYM].XXXXX)

6. Adaptations of single figures do not require NPG approval. However, the adaptation should be credited as follows:

   Adapted by permission from Macmillan Publishers Ltd: [JOURNAL NAME] (reference citation), copyright (year of publication)

   <u>Note: For adaptation from the *British Journal of Cancer*, the following credit line applies.</u>
   Adapted by permission from Macmillan Publishers Ltd on behalf of Cancer Research UK: [JOURNAL NAME] (reference citation), copyright (year of publication)

7. Translations of 401 words up to a whole article require NPG approval. Please visithttp://www.macmillanmedicalcommunications.com for more information.Translations of up to a 400 words do not require NPG approval. The translation should be credited as follows:

   Translated by permission from Macmillan Publishers Ltd: [JOURNAL NAME] (reference citation), copyright (year of publication).

   <u>Note: For translation from the *British Journal of Cancer*, the following credit line applies.</u>
   Translated by permission from Macmillan Publishers Ltd on behalf of Cancer Research UK: [JOURNAL NAME] (reference citation), copyright (year of publication)

We are certain that all parties will benefit from this agreement and wish you the best in the use of this material. Thank you.

Special Terms:

v1.1

If you would like to pay for this license now, please remit this license along with your payment made payable to "COPYRIGHT CLEARANCE CENTER" otherwise you will be invoiced within 48 hours of the license date. Payment should be in the form of a check

or money order referencing your account number and this invoice number RLNK501089026.
Once you receive your invoice for this order, you may pay your invoice by credit card. Please follow instructions provided at that time.

Make Payment To:
Copyright Clearance Center
Dept 001
P.O. Box 843006
Boston, MA 02284-3006

For suggestions or comments regarding this order, contact RightsLink Customer Support:customercare@copyright.com or +1-877-622-5543 (toll free in the US) or +1-978-646-2777.

Gratis licenses (referencing $0 in the Total field) are free. Please retain this printable license for your reference. No payment is required.
